# Karyotypic differentiation of populations of the common shrew *Sorex araneus* L. (Mammalia) in Belarus

**DOI:** 10.3897/CompCytogen.11i2.11142

**Published:** 2017-05-11

**Authors:** Yury M. Borisov, Iryna A. Kryshchuk, Helen S. Gaiduchenko, Elena V. Cherepanova, Svetlana V. Zadyra, Elena S. Levenkova, Dmitriy V. Lukashov, Victor N. Orlov

**Affiliations:** 1 Severtsov Institute of Ecology and Evolution, Russian Academy of Sciences, Leninskij Prosp. 33, 119071 Moscow, Russia; 2 Scientific and Practical Center for Bioresources, National Academy of Sciences of Belarus, Akademicheskaya St. 27, 220072 Minsk, Republic of Belarus; 3 Shevchenko Kiev National University, Educational–Scientific Center Institute of Biology, Kiev, 03187 Ukraine

**Keywords:** Chromosome races, Robertsonian translocations, chromosomal differentiation, *Sorex araneus*

## Abstract

The common shrews, *Sorex araneus* Linnaeus, 1758, inhabiting the territory of Belarus, are characterized by a significant variation in the frequency of Robertsonian (Rb) translocations. The frequency clines for translocations specific of three chromosome races: the West Dvina (*gm*, *hk*, *ip*, *no, qr*), Kiev (*g/m*, *hi*, *k/o*, *n*, *p*, *q*, *r*), and Białowieża (*g/r*, *hn*, *ik*, *m/p*, *o*, *q*) have already been studied in this territory. In this communication we report new data on polymorphic populations with Rb metacentrics specific of the Neroosa race (*go*, *hi*, *kr*, *mn*, *p/q*) in south-eastern Belarus, analyse the distribution of karyotypes in southern and central Belarus and draw particular attention to the fixation of the acrocentric variants of chromosomes in this area. The results show that certain Rb metacentrics specific of the Neroosa, West Dvina, Kiev, and Białowieża races (namely, *go* and *pq*; *ip*; *ko*; *hn* and *ik*, respectively) are absent in many polymorphic populations. Thus, the karyotypic differentiation of *S. araneus* in the studied area is determined by unequal spread of different Rb translocations and by fixation of acrocentric variants of specific chromosomes.

## Introduction

The common shrew, *Sorex araneus* Linnaeus, 1758, a species inhabiting Eurasia, is a model object for population genetic studies due to its exclusive chromosomal polymorphism (for review, see: Wójcik et al. 2002, Shchipanov and Pavlova 2016). Four metacentric autosomes (*af*, *bc*, *jl*, and *tu*) and sex chromosomes (XX in females and XY1Y2 in males) are characteristic of species *S. araneus* (acrocentric morphs of chromosome arms *j* and *l* sporadically occur in populations through the species area), while ten autosomal arms (*g*, *h*, *i*, *k*, *m*, *n*, *o*, *p*, *q*, and *r*) can be presented as acrocentrics or be fused as metacenetrics (Searle et al. 1991). The designations of chromosome arms are constant irrespective of their condition either as separate acrocentrics (*g*, *h*,﻿ ﻿*i*, *k*, *m*, *n*, *o*, *p*, *q*, *r*) or as arms of metacentrics (*gm*, *hk*,﻿ ﻿*ip*, *no*, *qr*).

A convenient methodic approach to describe the chromosomal polymorphism of *S. araneus* is a subdivision of populations into chromosome races. “A chromosome race of *Sorex araneus* is defined as a group of geographically contiguous or recently separated populations which share the same set of metacentrics and acrocentrics by descent” (Hausser et al. 1994). Chromosome races of *S. araneus* differ in the composition and numbers (one–five pairs) of metacentrics, which were formed by Robertsonian (Rb) translocations involving 10 pairs of acrocentric chromosomes, *g*, *h*, *i*, *k*, *m*, *n*, *o*, *p*, *q*, and *r* (Searle et al. 1991, Hausser et al. 1994). The karyotype with ten pairs of acrocentric autosomes *g–i*, *k*, *m–r* (number of autosomes in diploid set, 2NA, in this karyotype equals 28) is considered as initial in chromosomal evolution of *S. araneus* (Wójcik et al. 2002). Metacentrics formed from Rb translocations are referred to as “race-specific fused chromosomes” (or “race-specific metacentrics”) (Zima et al. 1996). Monomorphic karyotypes are characteristic of some races (all race-specific Rb translocations are fixed), while polymorphism for 2–5 translocations was revealed in majority of chromosome races (see list of Shchipanov and Pavlova 2016).

The fixation of Rb translocations may have occurred in isolated small-size populations, for example, in glacial refugia. The data on mtDNA polymorphism in some European species of small mammals, including species of the genus *Sorex*, testify to the existence of multiple glacial refugia in Mediterranean and central Europe (Bilton et al. 1998, Deffontaine et al. 2005).

In the postglacial period, the previously isolated populations which migrated from refugia came into contact and hybridized with each other. The width of hybrid zones depends on the degree of chromosomal differences between contacting races. When races that differ in the combination of chromosome arms (metacentrics with monobrachial homology) make contact, narrow hybrid zones (0.5–5 km) are formed (Banaszek 1994, Narain and Fredga 1996, Szałaj et al. 1996, Bulatova et al. 2011, Orlov et al. 2012). If races which come into contact have no metacentrics with monobrachial homologies, wide hybrid zones are formed, and the clinal variation in Rb metacentric frequency can stretch for 50–100 km (Lukáčová et al. 1994, Brünner et al. 2002, Zima et al. 2003).

Six chromosome races: Kiev, Bobruysk, Białowieża, Turov, West Dvina, and Borisov, are known in the territory of Belarus by present time (Mishta et al 2000, Borisov et al 2010, 2014). Three of them, the Kiev, Białowieża, and West Dvina races, have continuous distribution ranges beyond the territory of Belarus. The Kiev race (*g/m*, *hi*, *k/o*, *n*, *p*, *q*, *r*) (Mishta 1994) inhabits the western territory of the Ukraine, and this race was recently discovered in southern Belarus (Borisov et al. 2014, 2016). The distribution area of the Białowieża race (*g/r*, *hn*, *ik*, *m/p*, *o*, *r*; Fredga and Nawrin 1977) stretches from eastern Poland to western Belarus (Mishta et al., 2000; Borisov et al. 2016). The West Dvina race (*gm*, *hk*, *ip*, *no*, *qr*; Bulatova et al. 2002) occupies a vast territory from the Valdai Hills to the Smolensk Upland, its southern boundary passes through Vitebsk region of Belarus (Bulatova et al. 2002, Borisov et al. 2008, Orlov and Borisov 2009). The Borisov race (*g/m*, *h/k*, *i*, *n/o*, *q/r*, *p*; Orlov and Borisov 2009), which is a derivative of race West Dvina, is distributed in the middle Berezina basin (Borisov et al. 2010). The Bobruysk (*g*, *h/i*, *k*, *m*, *n*, *o*, *p*, *q*, *r*) and Turov (*g*, *h/k*, *i*, *m*, *n*, *o*, *p*, *q*, *r*) races were described in the vicinities of Bobruisk and Turov towns, respectively (Mishta et al. 2000).

The clinal variation in the frequencies of Rb metacentrics, similar to the clinal variation in wide hybrid zones, was observed in the polymorphic populations of the Kiev, Białowieża, West Dvina, and Borisov races in Belarus. Karyotypes with ten pairs of acrocentric chromosomes (*g*, *h*, *i*, *k*, *m*, *n*, *o*, *p*, *q*, *r*) were found in some of these polymorphic populations (Borisov et al. 2010, 2014). Such a pattern of karyotype distribution may be associated either with the selection against heterozygotes in the interracial hybrid zone (“acrocentric peak”) (term by J. Searle 1986) or with the spread of the Rb translocations of the metacentric races in populations characterized by ten pairs of acrocentric chromosomes (Borisov et al. 2014, 2016).

In this communication, we report new data on the distribution of Rb metacentrics specific of the Neroosa race (*go*, *hi*, *kr*, *mn*, *p/q*; Bulatova et al. 2000) in southeastern Belarus, analyse the distribution of the Białowieża, and Kiev races in southern and central Belarus and draw particular attention to the fixation of the acrocentric chromosomes in this area.

## Materials and methods

Animals were captured at seven sites within the low Pripyat and Dnieper River basins (Gomel’ and Mogilev regions) in July–September, 2014 and in September, 2015 (Table 1).

**Table 1.** Collection sites, chromosome races and karyotypes of common shrews in the territory of Belarus. The numbers indicate localities in Fig. 1. Polymorphism for Rb translocation is indicated by slash (/). Ne, Neroosa; Ki, Kiev; Bi, Białowieża; Wd, West Dvina; Bs, Borisov. S.s., sample size; ?, attribution to any race is unclear.

No.	Locality	Latitude; Longitude	S.s.	Race	2NA	Karyotypes
4	Dobrush	52°24'59"N; 31°17'12"E	**7**			
			1	Ne	23	*g*, *hi*, *k/r*, *mn*, *o*, *p*, *q*
			3	Ne	24	*g*, *hi*, *k/r*, *m/n*, *o*, *p*, *q*
			1	Ne	24	*g*, *hi*, *k*, *mn*, *o*, *p*, *q*, *r*
			2	Ne	26	*g*, *hi*, *k*, *m*, *n*, *o*, *p*, *q*, *r*
5	Gomel’	52°25'29"N; 30°52'31"E	**4**			
			1	Ne	23	*g*, *hi*, *k/r*, *mn*, *o*, *p*, *q*
			2	Ne	24	*g*, *hi*, *k/r*, *m/n*, *o*, *p*, *q*
	*		1	Ne	24	*g*, *hi*, *k*, *mn*, *o*, *p*, *q*, *r*
6	settl. Chernoye (Rechitsa distr.)	52°26'47"N; 30°22'50"E	**23**			
			4	Ne	24	*g*, *hi*, *k/r*, *m/n*, *o*, *p*, *q*
			9	Ne	25	*g*, *hi*, *k*, *m/n*, *o*, *p*, *q*, *r*
			9	?	26	*g*, *hi*, *k*, *m*, *n*, *o*, *p*, *q*, *r*
			1	Ki	25	*g*, *hi*, *k/o*, *m*, *n*, *p*, *q*, *r*
7	settl. Krasnoye (Bragin distr.)	51°33'50"N; 30°29'55"E	**14**			
			5	Ne		*g*, *hi*, *k/r*, *m/n*, *o*, *p*, *q*
			3	Ne	25	*g*, *hi*, *k/r*, *m*, *n*, *o*, *p*, *q*
			4	Ne	26	*g*, *hi*, *k*, *m*, *n*, *o*, *p*, *q*, *r*
			2	Ki	25	*g*, *hi*, *k/o*, *m*, *n*, *p*, *q*, *r*
18.3	Bobruisk	53°4'12"N; 29°14'28"E	**8**			
			2	Ki	25	*g*, *hi*, *k/o*, *m*, *n*, *p*, *q*, *r*
			2	Ki	26	*g*, *h/i*, *k/o*, *m*, *n*, *p*, *q*, *r*
			3	Ki	26	*g*, *hi*, *k*, *m*, *n*, *o*, *p*, *q*, *r*
			1	Ki	27	*g*, *h*, *i*, *k/o*, *m*, *n*, *p*, *q*, *r*
31	settl. Elizovo (Bobruisk distr.)	53°24'20"N; 29°0'30"E	**4**			
			1	Bi	25	*g*, *hn*, *i/k*, *m*, *o*, *p*, *q*, *r*
			1	Bi	25	*g*, *h/n*, *ik*, *m*, *o*, *p*, *q*, *r*
			2	Bi	26	*g*, *h/n*, *i/k*, *m*, *o*, *p*, *q*, *r*
32	settl. Lyubonichi (Bobruisk Distr.)	53°15'19"N; 29°10'21"E	**14**			
			2	Bi	25	*g*, *hn*, *i/k*, *m*, *o*, *p*, *q*, *r*
			2	Bi	25	*g*, *h/n*, *ik*, *m*, *o*, *p*, *q*, *r*
			5	Bi	26	*g*, *h/n*, *i/k*, *m*, *o*, *p*, *q*, *r*
			1	Bi	27	*g*, *h/n*, *i*, *k*, *m*, *o*, *p*, *q*, *r*
			2	Bi	27	*g*, *h*, *i/k*, *m*, *n*, *o*, *p*, *q*, *r*
			2	Bi	28	*g*, *h*, *i*, *k*, *m*, *n*, *o*, *p*, *q*, *r*

**Figure 1.**
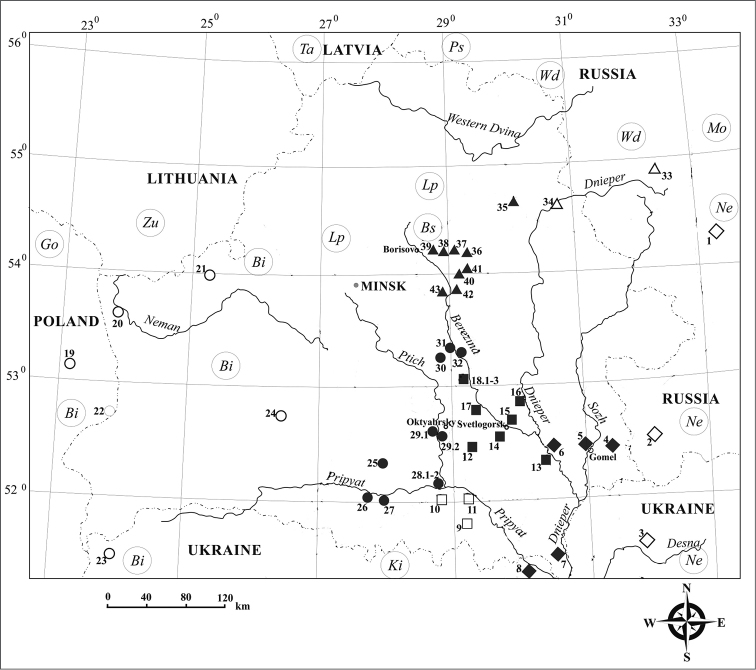
The distribution of the chromosome races of the common shrew in Belarus and neighbouring territories: Ne, Neroosa (diamonds); Ki, Kiev (squares); Bi, Białowieża (circles); Wd, West Dvina (light triangles); Bs, Borisov (black triangles); Go, Goldap; Zu, Zuvintas; Ta, Tallin; Ps, Pskov; Lp, Lepel; Mo, Moscow. See Suppl. material 1 for numbers of the collection sites. Dotted line indicates state borders.

The new material includes 75 *S. araneus* individuals (37 males and 38 females). Our karyological data on 290 shrews trapped in 2009–2013 (Borisov et al., 2010, 2014, 2016) and data on 252 shrews presented by other authors were used to determine the distribution range of race-specific metacentrics. In total, 603 individuals from 43 sites (an area from 51°22' to 54°55'N; from 23°10' to 34°10'E) have been analysed in this work (Suppl. material 1, Fig. 1). The study area is a mosaic of forest and meadow biotopes with occurrences of man-made landscapes.

Chromosome preparations were obtained from bone marrow and spleen cells after a routine technique with colchicine treatment (Ford and Hamerton 1956). Chromosome identification was carried out by G-banding method with trypsin (Seabright 1971) in accordance with the international common shrew chromosome nomenclature (Searle et al. 1991).

## Results

Karyotyping of *S. araneus* individuals captured in the southeastern territory of Belarus (at three sites to the east and one site to the west of the Dnieper River) helped to identify three Rb metacentrics, *hi*, *kr* and *mn* (Table 1, Fig. 1, nos. 4–7). These metacentrics are specific of the Neroosa race (*go*, *hi*, *kr*, *mn*, *p/q*); however, other two metacentrics of this race, *go* and *pq*, have not been found at the sites studied. The Rb translocation *hi* is fixed in all the examined samples, and chromosome arms *k*, *r* and *m*, *n* occur both as metacentric and acrocentric morphs (Table 1). The *kr* translocation was only found in heterozygous condition. Karyotypes with metacentric *ko* which is characteristic of the Kiev race (*g/m*, *hi*, *k/o*) were found in two samples from Rechitsa and Bragin districts (nos. 6 and 7).

In karyotypes of eight shrews captured near Bobruisk town (the west bank of the Berezina River), two Rb metacentrics of the Kiev race (*g/m*, *hi*, *k/o*), *hi* and *ko*, were observed (Table 1, Fig. 1, no. 18.3). The *hi* translocation appeared both in homozygous and heterozygous condition, and the *ko* translocation – only in heterozygous condition. Metacentric *gm* which is characteristic of the Kiev race was not revealed in our sample.

At two sites to the north of Bobruisk, on the west and east banks of the Berezina, karyotypes with two metacentrics of the Białowieża race (*g/r*, *hn*, *ik*, *m/p*), *hn* and *ik*, were found (Table 1, Fig. 1, nos. 31, 32). Heterozygotes for the *hn* and *ik* translocations prevailed in the samples. Metacentrics *gr* and *mp*, specific of this chromosome race, were not revealed.

## Discussion

The new results of the karyological study of *S. araneus* populations in eastern Belarus together with previously published data show a considerable variation in the frequency of Rb metacentrics characteristic of the Neroosa race in this area and the closest territories. The Neroosa race (*go*, *hi*, *kr*, *mn*, ﻿and *p/q*; Bulatova et al. 2000) is monomorphic for four Rb translocations throughout its large distribution range in the Oka and Don River basins (Bystrakova et al. 2007). The acrocentric chromosomes *g*, *o*, *k*, and *r* were noted at some sites in Ukraine (Mishta et al. 2000) and near the Belarusian boundary, in the vicinity of Novozybkov city (see Suppl. material 1) where 2NA varied from 19 to 25 (Bulatova et al. 2000, Sheftel and Krysanov 2002). It is supposed that homozygotes for acrocentric morphs *g*, *o* and *p*, *q* existed among individuals 2NA=25 described by Sheftel and Krysanov (2002) (unfortunately, the data on individual karyotypes were not shown). However, the registered karyotypes with 2NA=19 testify to the presence of all metacentrics specific of the Neroosa race in populations from Novozybkov. As for populations in the Gomel region, only one Rb translocation, *hi*, is fixed; chromosome arms *k*, *r* and *m*, *n* are presented by acrocentric and metacentric morphs, while arms *g*, *o* and *p*, *q* are presented exclusively by acrocentric morphs (Tables 1, 2, nos. 4–7).

A significant variation in the Rb translocation frequency was earlier described in populations of chromosome races West Dvina, Borisov, Kiev, and Białowieża in the Dnieper–Pripyat interfluve (Orlov and Borisov 2009, Borisov et al. 2010, Borisov et al. 2014, 2016). It should be noted that the West Dvina race (*gm*, *hk*, *ip*, *no*,﻿ ﻿*qr*; Bulatova et al. 2002), like the Neroosa race, is monomorphic for five Rb translocations throughout most part of its distribution range, from the Valdai Hills to the Smolensk Upland (Borisov et al. 2008). Acrocentric morphs of chromosomes *n*, *o* and *q*, *r* were found to the south of the Smolensk Upland (Fig. 1, no. 34; see Suppl. material 1). The Borisov race (a derivative of the West Dvina race) inhabiting the territory along the Berezina River (nos. 35–39) is polymorphic for the *gm*, *hk*, *no*, and *qr* translocations; however, chromosomes *i* and *p* appear only as acrocentrics (Orlov and Borisov 2009, Borisov et al. 2010). Karyotypes with two Rb translocations, *gm* and *hk*, and karyotypes with ten pairs of acrocentrics (*g*, *h*,﻿ ﻿*i*, *k*, *m*, *n*, *o*, *p*, *q*, *r*) were revealed in populations southwards of the town of Borisov (Table 2, nos. 40–43). The analysis of additional samples is required to determine whether these polymorphic populations may be attributed to the Borisov race.

**Table 2.** Frequencies of race-specific metacentrics in populations of eight chromosome races of *S. araneus*. Numbers of sites are the same as in Table 1 and in Fig. 1; the frequency of Rb translocations is an average of studied specimens from all samples. S.s., sample size; two-three digits in column “S.s”. are arranged in the same order as references in column “References”.

Nos, Sites	References	S.s.	Metacentric frequencies				
			**race Neroosa**				
			*go*	*hi*	*kr*	*mn*	*pq*
1: Spas-Demensk	Bulatova et al. 2000	6	1.0	1.0	1.0	1.0	1.0
2: Novozybkov	Bulatova et al. 2000	3	0.33	1.0	1.0	0.66	0.50
3. Berezna	Mishta et al. 2000	2	1.0	1.0	1.0	1.0	1.0
4–6: Dobrush, Gomel, Chernoye	new data	34	0.0	1.0	0.17	0.39	0.0
7: Krasnoye	new data	14	0.0	1.0	0.13	0.21	0.0
			**race Kiev**				
			*gm*	*hi*	*ko*		
9–11: Yeslk, Leshnya, Mozyr’	Borisov et al. 2014, 2016	2, 29	0.08	0.65	0.16		
12, 13: Ozarichi, Rechitsa	Borisov et al. 2014, 2016	3, 16	0.0	0.72	0.53		
14, 15: Sosnovyi Bor, Svetlogorsk	Borisov et al. 2016	10	0.0	0.65	0.15		
16, 17: Zhlobin Parichi	Borisov et al. 2016	25	0.0	0.50	0.19		
18.1–18.3: Bobruisk)	Borisov et al. 2016, Mishta et al. 2000, new data	2, 1, 8	0.0	0.67	0.33		
			**race Białowieża**				
			*gr*	*hn*	*ik*	*mp*	
19: Bialystok	Banaszek et al. 2009	56	0.91	1.0	1.0	0.71	
20: Grodno	Borisov et al. 2014	2	1.0	1.0	1.0	1.0	
21: Lesnoe Ozero	Mishta et al. 2000	5	0.20	1.0	1.0	0.30	
22: Białowieża	Wójcik et al. 1996	87	0.99	1.0	1.0	0.95	
24: Ganzevichi	Borisov et al. 2014	2	0.50	1.0	1.0	0.50	
25: Chervonoye	Borisov et al. 2014	15	0.03	0.67	0.50	0.05	
26, 27: Turov, Khvoyensk	Borisov et al. 2014, 2016	23, 21	0.01	0.44	0.58	0.00	
29: Oktiabr’skiy	Borisov et al. 2014, 2016	22, 19	0.01	0.33	0.37	0.01	
30–32: Tatarka, Elizovo, Lyubonichi Borisov et al. 2014, 2016, new data	Borisov et al. 2014, 2016, new data	14, 18	0.0	0.71	0.56	0.0	
			**race West Dvina**				
			*gm*	*hk*	*ip*	*no*	*qr*
33: Kardymovo	Orlov Borisov 2009	2	1.0	1.0	1.0	1.0	1.0
34: Dubrovno	Orlov, Borisov 2009	3	0.5	1.0	0.75	1.0	0.75
			**race Borisov**				
			*gm*	*hk*	*ip*	*no*	*qr*
35: Smolyany	Orlov, Borisov 2009	2	0.5	1.0	0.0	1.0	0.5
36–39: Malyi Vyazok – Novaya Metcha	Orlov, Borisov 2009, Borisov et al. 2010	5, 33	0.84	0.99	0.0	0.38	0.09
			**Polymorphic populations of unclear attribution**				
40, 41: Leskovichi Mikhevichi	Borisov et al. 2010	7	0.64	0.86	0.0	0.0	0.0
42, 43: Berezino, Yedlino	Orlov, Borisov 2009, Borisov et al. 2010	2, 27	0.02	0.59	0.0	0.0	0.0

**Figure 2.**
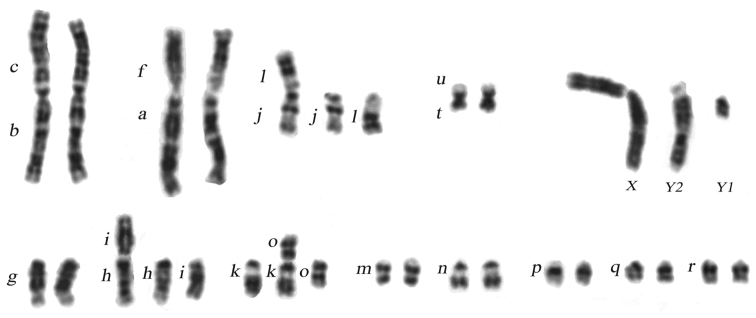
G-banded karyotype of male shrew of the Kiev race (Svetlogorsk vic., Belarus), *g, h/i, j/l, k/o, m, n, o, p, q, r* (2NA=26).

In the populations to the west of the Dnieper River, in the lower part of the Berezina River basin, two metacentrics of the Kiev race (*g/m*, *hi*, *k/o*, Mishta 1994), namely, *hi* and *ko* (Fig. 2), are distributed (Table 2, Fig. 1, nos. 12–18); metacentric *gm* was not found (Borisov et al., 2016). All three Rb metacentrics specific of the Kiev race were revealed on the south bank of the Pripyat (nos. 9 - 11), however, the frequency of the *gm* metacentric occurred to be lower than in the samples from the Ukraine (Mishta et al. 2000). Taking into account the new data, we note that distribution area of Rb metacentrics *hi* and *ko* includes type locality of the Bobruysk race (*g*, *h/i*, *k*, *m*, *n*, *o*, *p*, *q*, *r*; Mishta et al. 2000) (Suppl. material 1, nos 18.1 – 18.3). Hence, the individuals carrying a single Rb translocation *hi* may be regarded as representatives of polymorphic race Kiev.

The shrews with Rb metacentrics of the Neroosa race (*kr* and *mn*) and the shrews with metacentric of the Kiev race (*ko*) were found in samples from Rechitsa and Bragin districts, and hybrid individuals with metacentrics of both the races (simple heterozygotes *hi*,﻿ ﻿*k/o*, *m/n*) were found in the vicinity of Rechitsa city (Suppl. material 1, nos. 6 and 7, and 13). Thus, the hybrid zone between the polymorphic populations of the Neroosa and Kiev races approximately passes along the Dnieper. Racial attribution of homozygotes for the *hi* translocation from Rechitsa and Bragin districts is unclear (Fig. 1, nos. 6 and 7).

The polymorphism for Rb translocations was earlier detected in the *S. araneus* population (a sample of 14 individuals; Table 1, no. 8) from the neighbourhood of Chernobyl; 2NA varied from 24 to 26 (Baker et al. 1996). The chromosomes were not identified by differential G-banding; the animals were not affiliated with any race. Previously known data of Bulatova et al. (2000) and Mishta et al. (2000) and our new results (Suppl. material 1, Fig. 1, nos. 2, 3, 6, 7, 9, 13) suggest that both the shrews of the Neroosa and shrews of the Kiev races, may live in the neighbourhood of Chernobyl’. It cannot be excluded that the population at site near Chernobyl belongs to the contact zone of these chromosome races.

In the populations inhabiting the southwestern territory of Belarus along the Ptich River and at some sites of the south bank of the Pripyat River, the metacentrics of the Białowieża race (*g/r*, *hn*, *ik*, *m/p*, Fredga and Nawrin 1977), *hn* and *ik*, were observed (Fig. 1, nos. 25–32; see Suppl. material 1). As for the *gr* and *mp* metacentrics, they were only revealed at three of the mentioned sites (frequency did not exceed 0.05; Table 2, nos. 26, 27, 29) and were not found at four sites (nos. 15, 30–32). For comparison, in eastern Poland, the *hn* and *ik* translocations are fixed, and the minimal frequencies of the *gr* and *mp* translocations are equal to 0.91 and 0.71, respectively (Table 2, 19, 22) (Wójcik et al. 1996, Banaszek et al. 2009).

Hybrid individuals with Rb metacentrics of the Kiev and Białowieża races: simple heterozygotes (*hn*, *ko; g/m*, *h/n, i/k*) and complex heterozygotes (metacentrics with monobrachial homology: *i/hi/hn/n* and *i/ik/ko/o*, are present in their karyotypes), were revealed along the Ptich River and on the south bank of the Pripyat River, close to the confluence of the Pripyat and Uborot’ Rivers (Suppl. material 1, nos. 10, 26, 27, and 29). The contact and hybrid zone between these races extends along the Ptich River and continues on the south bank of the Pripyat River. Karyotypes with ten pairs of acrocentrics were observed in some polymorphic populations of the Kiev and Białowieża races (Suppl. material 1, nos. 16, 17, 26–29.2, and 32).

There are two possible explanations of chromosome variation and a high frequency of acrocentrics in *S. araneus* populations in Belarus.

1) Hybridization between metacentric races which differ for the arm combinations of Rb metacentrics (*e.g.* chromosome arms *g*, *h*, *i*, *k*, *m*, *n*, *o*, *p*, *r* are combined as metacentrics *gr*, *hn*, *ik*, *mp* in karyotype of the Białowieża race and as metacentrics *gm*, *hi*, *ko* in the karyotype of the Kiev race). The low fitness of hybrids (complex heterozygotes possessing metacentrics with monobrachial homology, *e.g. r/gr/gm/mp/p*,﻿ ﻿*n/hn/hi/ik/ko/o*) leads to the decrease of metacentric frequency. This phenomenon is called “acrocentric peak” (Searle 1986).

2) Hybridization between metacentric races and an acrocentric race that existed in the Dnieper basin in the past (Borisov et al. 2016).

Our hypothesis about the existence of acrocentric race in the present-day Belarus or neighbouring territory during the Last glacial maximum (LGM, 24–17 kyr BP) does not contradict the paleontological and paleobotanic data: fossil remains of the common shrew were found in the Middle Dnieper basin (Markova and Pusachenko 2008) and a forest refugium of LGM was revealed in this territory (Simakova and Pusachenko 2008). Proceeding from the data on the current distribution range of chromosome races and the data on the locations of Late Pleistocene forest refugia (Kozharinov 1994), we may come to the conclusion that the West Dvina, Neroosa, and Kiev races survived the most recent glacial period in the refugia of the Valdai Hills, the Middle Russian Upland, and the Carpathians (Orlov et al. 2008).

The karyotypic differentiation of *S. araneus* in the low Dnieper and Pripyat basin is determined by unequal spread of different Rb translocations and by fixation of acrocentric variants of the particular chromosome arms. Each of four groups of the polymorphic populations possessing metacentrics, which are specific of the Neroosa, West Dvina, Kiev, and Białowieża races, consists of two subgroups: 1) polymorphic populations with all Rb metacentrics of the initial race, irrespective of their frequencies (Fig. 1, light figures); 2) polymorphic populations which lack 1-2 race-specific Rb metacentrics (Fig. 1, black figures). It should be stressed that the extent of distribution areas for the populations with fixed acrocentric morphs of definite chromosome arms exceeds 50 kilometres.

The subdivision of the populations of the *S. araneus* into chromosome races is a simplified methodic approach to describe the chromosomal polymorphism of this species (only the presence / absence of any Rb translocation is taken into consideration irrespective of its frequency; see Searle et al. 2003). According to the definition of a chromosome race as “ … populations which share the same set of metacentrics and acrocentrics by descent” (Hausser et al. 1994), the populations that differ from neighbouring population owing to the presence or absence of a Rb translocation and occupy a definite area may be regarded as separate chromosome races. For example, the populations in Sweden that differ from the Abisko race (*g/m*, *h/n*, *i/p*, *k/q*, and *o/r*) by acrocentric variant of the arms *o* and *r* were recognized as the Ammarnas and Hattsjo races (Fredga 2007), and the population in the Rügen Island that differs from the mainland Danish race Jutland (*gm*, *hi*, *kq*, *no*) by acrocentric variant of arms *n* and *o* was recognized as the Rügen race (Brünner et al. 2002). Thus, the populations in the Dnieper – Pripyat interfluve which lack 1-2 race-specific Rb metacentrics, may be regarded as new chromosome races.

(1) The absence of metacentrics *go* and *pq*, that is, the fixation of acrocentric variants *g*, *o*, and *p*, *q* in *S. araneus* populations from the low Dnieper, Sozh and Pripyat basin (a total of 39 individuals from four sites; Tables 1 and 2, Fig. 1, nos. 4–7) allows them to be recognized as a race of its own called “Gomel’ ’’.

Chromosome race: Gomel’ (Gm)

Karyotype: XX/XY1Y2, *af*, *bc*, *g*, *hi*, *jl*, *k/r*, *m/n*, *o*, *p*, *q*, *tu*

Type locality: vicinity of Gomel’ city, Belarus, 52°25'29"N, 30°52'31"E.

Distribution range: An area between Dnieper and Sozh Rivers, Gomel, and Rechitsa district; to the south of Rechitsa city to the latitude of Bragino city. The western boundary of the range lies along the west bank of the Dnieper River, approximately at the longitude of Rechitsa city. The other boundaries are not determined.

(2) All the three Rb metacentrics characteristic of the Kiev race occur only in populations to the south of the Pripyat River (Table 2, Fig. 1, nos. 9–11). We suppose that the polymorphic populations of the common shrew inhabiting the area between Dnieper and Pripyat Rivers (a total of 65 individuals from eight sites; Tables 1 and 2, Fig. 1, nos. 12–18), with fixed acrocentric morphs *g* and *m*, can be recognized as the Svetlogorsk race.

Chromosome race: Svetlogorsk (Sv)

Karyotype: XX/XY1Y2, *af*, *bc*, *g*, *h/i*, *j/l*, *k/o*, *m*, *n*, *p*, *q*,﻿ ﻿*r*, *tu* (see Fig. 2).

Type locality: vicinity of Svetlogorsk city, Belarus, 52°31'46"N, 29°34'49"E.

Distribution range: an area between Dnieper and Pripyat Rivers (Belarus, Gomel region); the low Berezina basin to the vicinity of Parichi and Zhlobin cities to the north, from the east bank of the Ptich River and to the west bank of the Dnieper River.

(3) The absence of metacentrics *gr* and *mp*, specific of the Białowieża race, in *S. araneus* populations inhabiting the territory along the Ptich River allows us to recognize these populations (a total of 104 individuals from nine sites) (Table 2; Fig. 1, nos. 25–27 and 29–32) as the Oktiabr’skiy race.

Chromosome race: Oktiabr’skiy (Ok)

Karyotype: XX/X Y1Y2, *af*, ﻿*bc*, *g*, *h/n*, *j/l*, *i/k*, *m*, *o*, *p*, *q*, *r*

Type locality: Rozhanov settlement, vicinity of Oktiabr’skiy town, Belarus 52°34'26"N, 28°44'37"E.

Distribution range: Southwestern Belarus, territory along the Ptich River, approximately to the latitude of Osipovichi city to the north. The western boundary is not determined. Easternmost site for shrews of this race is on the east bank of the Berezina River. The southern boundary extends along the south bank of the Pripyat River from Turov city to the confluence of the Ptich and Pripyat Rivers.

Now, 74 chromosome races of the common shrew (including 49 polymorphic ones) are known (Shchipanov and Pavlova 2016), however, the study of chromosomal polymorphism of this species is not yet completed. Equally with molecular-genetic and morphometric data, information on distribution and fixation of different Rb translocations in definite parts of the *S. araneus* range is very important for study of intraspecies structure of the *S. araneus*. Unequal spread of different Rb translocations and fixation of acrocentric variants of particular chromosome arms observed in each of these groups is an interesting example of karyotypic differentiation in populations of the common shrew.

## Supplementary materials

Supplementary material 1 Collection sites, chromosome races and karyotypes of common shrews in the Dnieper and Pripyat river basins (Belarus) and neighboring areas Data type: occurence This dataset is made available under the Open Database License (http://opendatacommons.org/licenses/odbl/1.0/). The Open Database License (ODbL) is a license agreement intended to allow users to freely share, modify, and use this Dataset while maintaining this same freedom for others, provided that the original source and author(s) are credited.Yury M. Borisov, Iryna A. Kryshchuk, Helen S. Gaiduchenko, Elena V. Cherepanova, Svetlana V. Zadyra, Elena S. Levenkova, Dmitriy V. Lukashov, Victor N. Orlov

## References

[B1] Baker RJ Van Den Bussche RA Wright AJ Wiggins LE Hamilton MJ Reat EP Smith MH Lomakin MD Chesser RK (1996) High levels of genetic change in rodents of Chernobyl. Nature 380(6576): 707–708. https://doi.org/10.1038/380707a0 8614463 10.1038/380707a0

[B2] Banaszek A (1994) The structure of the contact zone between the chromosome races Druzno and Łegucki Młyn in the common shrew (*Sorex araneus*) in north-eastern Poland. Folia Zoologica 43(Suppl 1): 11–19.

[B3] Banaszek A Taylor JRE Ochocińska D Chętnicki W (2009) Robertsonian polymorphism in the common shrew (*Sorex araneus* L.) and selective advantage of heterozygotes indicated by their higher maximum metabolic rates. Heredity 102: 155–162. https://doi.org/10.1038/hdy.2008.102 18827836 10.1038/hdy.2008.102

[B4] Bilton DT Mirol PM Mascheretti S Fredga K Zima J Searle JB (1998) Mediterranean Europe as an area of endemism for small mammals rather than a source for northwards postglacial colonization. Proceedings of Royal Society London B 265(1402): 1219–1226. https://doi.org/10.1098/rspb.1998.0423 10.1098/rspb.1998.0423 PMC1689182 9699314

[B5] Borisov YuM Kozlovsky AI Balakirev AE Demidova TB Irchin SYu Malygin VM Okulova NM Potapov SG Shchipanov AN Orlov VN (2008) Contact zones between chromosome races of the common shrew *Sorex araneus* L. (Insectivora, Mammalia) at the margins of the Veps Stage of the Valdai ice sheet. Sibirskii Ekologicheskii Zhurnal 15(5): 763–771. https://doi.org/10.1134/s1995425508050111 [In Russian with English translation in Contemporary Problems of Ecology 1(5): 583–589]

[B6] Borisov YuM Cherepanova EV Orlov VN (2010) A wide hybrid zone of chromosome races of the common shrew, *Sorex araneus* Linnaeus, 1758 (Mammalia), between the Dnieper and Berezina Rivers (Belarus). Comparative Cytogenetics 3(2): 195–201. https://doi.org/10.3897/compcytogen.v4i2.43

[B7] Borisov YuM Kryshchuk IA Cherepanova EV Gajduchenko HS Orlov VN (2014) Chromosomal polymorphism of populations of the common shrew, *Sorex araneus* L., in Belarus. Acta Theriology 59(2): 243–249. https://doi.org/10.1007/s13364-013-0160-y

[B8] Borisov YuM Gaiduchenko HS Cherepanova EV Kryshchuk IA Nikiforov ME Orlov VN (2016) The clinal variation of metacentric frequency in the populations of the common shrew, *Sorex araneus* L., in the Dnieper and Pripyat interfluve. Mammal Research 61(2): 269–277. https://doi.org/10.1007/s13364-016-0272-2

[B9] Brünner H Turni H Kapischke HJ Strubbe M Vogel P (2002) New *Sorex araneus* karyotypes from Germany and the postglacial recolonization of Central Europe. Acta Theriologica 47(3): 277–293. https://doi.org/10.1007/BF03194147

[B10] Bulatova NSh Searle JB Bystrakova N Nadjafova R Shchipanov N Orlov V (2000) The diversity of chromosome races in *Sorex araneus* from European Russia. Acta Theriologica 45(Suppl 1): 33–46. https://doi.org/10.4098/AT.arch.00-60

[B11] Bulatova NSh Nadjafova RS Krapivko TP (2002) Intraspecific phylogenetic relationships in *Sorex araneus* L.: the Southern Baltic Subgroup of chromosome races. Russian Journal of Genetics 38(1): 64–69. https://doi.org/10.1023/A:1013767828674 [In Russian with English translation in Genetika 38(1): 79–85] 11852798

[B12] Bulatova NSh Jones RM White TA Shchipanov NA Pavlova SV Searle JB (2011) Natural hybridization between extremely divergent chromosome races of the common shrew (*Sorex araneus*, Soricidae, Soricomorpha): hybrid zone in European Russia. Journal of Evolutionary Biology 24: 573–586. https://doi.org/10.1111/j.1420-9101.2010.02191.x 21159004 10.1111/j.1420-9101.2010.02191.x

[B13] Bystrakova N Shchipanov NA Bulatova N Sheftel BI Nadjafova RS Pavlova SV Demidova TB Bobretsov AB Alexandrov DYu Kalinin AA Kouptsov AV Volkova AT Oleinichenko VYu Searle JB (2007) New data on the geographic distribution of chromosome races of *Sorex araneus* (Soricidae, Eulipotyphla) in European Russia. Russian Journal of Theriology 6(1): 105–109.

[B14] Deffontaine V Libois R Kotlik P Sommer R Nieberding C Paradis E Searle JB Michaux JR (2005) Beyond the Mediterranean peninsulas: evidence of central European glacial refugia for a temperate forest mammal species, the bank vole (*Clethrionomys glareolus*). Molecular Ecology 14: 1727–1739. https://doi.org/10.1111/j.1365-294X.2005.02506.x 15836645 10.1111/j.1365-294X.2005.02506.x

[B15] Ford CE Hamerton JL (1956) A colchicine hypothonic citrate, squash sequence for mammalian chromosomes. Stain Technology 31: 247–251. https://doi.org/10.3109/10520295609113814 13380616 10.3109/10520295609113814

[B16] Fredga K (2007) Reconstruction of the postglacial colonization of *Sorex araneus* into northern Scandinavia based on karyotype studies, and the subdivision of the Abisko race into three. Russian Journal of Theriology 6(1): 85–96.

[B17] Fredga K Nawrin J (1977) Karyotype variability in *Sorex araneus* L. (Insectivora, Mammalia). Chromosomes Today 6: 153–161.

[B18] Hausser J Fedyk S Fredga K Searl JB Volobouev V Wójcik JM Zima J (1994) Definition and nomenclature of the chromosome races of *Sorex araneus*. Folia Zoologica 43(Suppl 1): 1–9.

[B19] Kozharinov AV (1994) Vegetation dynamic of Eastern Europe in late glaciation – Holocene. Autoreferat of Ph.D. Dissertation, Moscow, Russian Federation: Institute of Ecology and Evolution, Russian Academy of Sciences, 47 pp. [In Russian]

[B20] Lukáčová L Pialec J Zima J (1994) A hybrid zone between the Ulm and Drnholec karyotypic races of *Sorex araneus* in the Czech Republic. Folia Zoologica 43(Suppl 1): 37–42.

[B21] Markova AK Pusachenko AYu (2008) Mammal assembles of the Last Glacial Maximum (LGM, 24 – 17 kyr. BP). In: Markova A Van Kolfschoten T (Eds) Evolution of the European Ecosystems during Pleistocene-Holocene Transition (24 – 8 kyr. BP). KMK Scientific Press, Moscow, 91–116. [In Russian, with English summary]

[B22] Mishta A (1994) A karyological study of the common shrew, *Sorex araneus* L. (Insectivora, Soricidae) in Ukraine: first results. Folia Zoologica (Brno) 43(Suppl 1): 63–68.

[B23] Mishta AV Searle JB Wójcik JM (2000) Karyotypic variation of the common shrew *Sorex araneus* in Belarus, Estonia, Latvia, Lithuania and Ukraine. Acta Theriology 45(Suppl 1): 47–58. https://doi.org/10.4098/AT.arch.00-61

[B24] Narain Y Fredga K (1996) A hybrid zone between the Hallefors and Uppsala chromosome races of *Sorex araneus* in central Sweden. Hereditas 125(2-3): 137–145. https://doi.org/10.1111/j.1601-5223.1996.00137.x

[B25] Orlov VN Borisov YuM (2009) Phylogenetic relationships between the common shrew (*Sorex araneus* L., Insectivora) populations in Belarus according to karyological data. Zoologichesky Zhurnal 88(12): 1506–1514. [In Russian, with English summary]

[B26] Orlov VN Kozlovskii AI Balakirev AE Borisov YuM (2008) Fixation of metacentric chromosomes in populations of the common shrew, *Sorex araneus* L., from Eastern Europe. Russian Journal of Genetics 44(5): 581–593. [Original Russian article: Genetika (2008) 44(5): 501–512]. PMID: 18672791. 18672791

[B27] Orlov VN Borisov YuM Cherepanova EV Grigor’eva OO Sycheva VB (2012) Narrow hybrid zone between Moscow and Western Dvina chromosome races and specific features of population isolation in common shrew *Sorex araneus* (Mammalia). Russian Journal of Genetics 48(1): 70–78. [Original Russian article: Genetika 48(1): 80–88]. PMID: 22567857. 22567857

[B28] Seabright M (1971) A rapid banding technique for human chromosomes // lancet 11: 971– 972. 10.1016/s0140-6736(71)90287-x 4107917

[B29] Searle J (1986) Factors responsible for a karyotypic polymorphism in the common shrew, *Sorex araneus*. Proceedings of Royal Society of London B 229: 277–298. https://doi.org/10.1098/rspb.1986.0086 10.1098/rspb.1986.0086 2881304

[B30] Searle JB Fedyk S Fredga K Hausser J Volobouev VT (1991) Nomenclature for the chromosomes of the common shrew (*Sorex araneus*). Memoires de la Societe Vaudoise des Sciences Naturelles. 19: 13–22. [Reprinted in: Comparative Cytogenetics (2010) 47(1): 87–96 . https://doi.org/10.3897/compcytogen.v4i1.28]

[B31] Shchipanov NA Pavlova SV (2016) Multi-level subdivision in the species group “*araneus*” of the genus *Sorex* . 1. Chromosomal differentiation. Zoologicheskii Zhurnal 95(2): 216–233. [In Russian with English summary]

[B32] Sheftel BI Krysanov EY (2002) Chromosome polymorphism of the Neroosa race (*Sorex araneus*) in the territory with radioactive pollution after the Chernobyl accident. In: Volobuev V (Ed.) 6-th Meeting of the International Sorex araneus Cytogenetics Committee (ISACC). Paris, September 3-7, 2002, 29–30.

[B33] Simakova A Puzachenko A (2008) The vegetation during the Last Glacial Maximum (LGM) (24.0–17.0 kyr BP) In: Markova A Van Kolfschoten T (Eds) Evolution of the European Ecosystems during Pleistocene-Holocene Transition (24–8 kyr. BP). KMK Scientific Press, Moscow, 315–341. [In Russian, with English summary]

[B34] Szałaj KA Fedyk S Banaszek A Chętnicki W Ratkiewicz M (1996) A hybrid zone between two chromosome races of the common shrew, *Sorex araneus*, in eastern Poland. Preliminary results. Hereditas 125: 169–176. https://doi.org/10.1111/j.1601-5223.1996.00169.x

[B35] Wójcik JM Borodin PM Fedyk S Fredga K Hausser J et al (2003) The list of chromosome races of the common shrew, *Sorex araneus* (updated 2002). Mammalia 68: 169–179. https://doi.org/10.1515/mamm.2003.67.2.169

[B36] Wójcik JM Ratkiewicz M Searle JB (2002) Evolution of the common shrew *Sorex araneus*: chromosomal and molecular aspects. Acta Theriologica 47(Suppl 1): 139–167. https://doi.org/10.1007/BF03192485

[B37] Wójcik JM Wójcik AM Zalewska H (1996) Chromosome and allozyme variation of the common shrew, *Sorex araneus*, in different habitats. In: Fredga K Searl JB (Eds) Evolution in the Sorex araneus Group. Cytogenetic and Molecular aspects. Proc of the ISAACC’s 5th Intern Meeting. Hereditas, Offprint 125: 183–189. https://doi.org/10.1111/j.1601-5223.1996.00183.x

[B38] Zima J Fedyk S Fredga K Hausser J Mishta A Searle JB Volobouev VT Wójcik JM (1996) The list of the chromosome races of the common shrew (*Sorex araneus*). In: Fredga K Searl JB (Eds) Evolution in the Sorex araneus Group. Cytogenetic and Molecular aspects. Proc of the ISAACC’s 5th Intern Meeting. Hereditas, Offprint 125: 97–107. https://doi.org/10.1111/j.1601-5223.1996.00097.x

[B39] Zima J Slivková L Tomášková L (2003) New data on karyotypic variation in the common shrew, *Sorex araneus*, from the Czech Republic: an extension of the range of the Laska race. Mammalia 68(2): 209–215. https://doi.org/10.1515/mamm.2003.67.2.209

