# Supplementary material for: Karyotypic differentiation of populations of the common shrew Sorex araneus L. (Mammalia) in Belarus
Source: Comp Cytogenet. 2017 May 11;11(2):359–73. doi: 10.3897/CompCytogen.11i2.11142 (PMC5596991; doi:10.3897/CompCytogen.11i2.11142)
Supplement: Supplementary material 1 — Collection sites, chromosome races and karyotypes of common shrews in the Dnieper and Pripyat river basins (Belarus) and neighboring areas [file comparative_cytogenetics-11-359-s001.doc]

**Supplemental Table.** Collection sites, chromosome races and karyotypes of common shrews in the Dnieper and Pripyat river basins (Belarus) and neighboring areas. The numbers indicate localities in Fig. 1. Polymorphism for Rb translocation is indicated by slash (/). S.s., sample size; 10a, karyotypes with ten acrocentric pairs; ?, attribution to any race is unclear.

| **No.** | **Locality** | **Latitude; Longitude** | **S.s.** | **Races** | **2NA** | **Karyotypes** | **Reference** |
| --- | --- | --- | --- | --- | --- | --- | --- |
| 1 | Spas-Demensk | 54°20'N; 34°10E' | **6** | Ne |  | *go, hi, kr, mn, pq* | Bulatova et al. 2000 |
| 2 | Novozybkov | 52°30'N; 32°20'E | **3** |  |  |  | Bulatova et al. 2000 |
|  |  |  |  | Ne | 21 | *go, hi, k/r, mn, p, q* |  |
|  |  |  |  | Ne | 23 | *g, hi, k/r, mn, o, p, q* |  |
|  |  |  |  | Ne | 24 | *g, hi, k, r, mn, o, p, q,* |  |
|  | Novozybkov | 52°30' N; 32°20 E' | **77** | Ne | 19-25 | *g/o, hi, k/r, m/n, p/q* | Sheftel, Krysanov 2002 |
| 3 | Berezna | 51°22' N; 32°09' E | **2** | Ne | 18 | *go, hi, kr, mn, pq* | Mishta et al. 2000 |
| 4 | Dobrush | 52°24'59"N; 31°17'12"E | **7** |  |  |  | new data |
|  |  |  | 1 | Gm | 23 | *g, hi, k/r, mn, o, p, q* |  |
|  |  |  | 3 | Gm | 24 | *g, hi, k/r, m/n, o, p, q* |  |
|  |  |  | 1 | Gm | 24 | *g, hi, k, mn, o, p, q, r* |  |
|  |  |  | 2 | Gm | 26 | *g, hi, k, m, n, o, p, q, r* |  |
| 5 | Gomel' | 52°25'29"N; 30°52'31"E | **4** |  |  | *g, hi, k/r, m/n, o, p, q* | new data |
|  |  |  | 1 | Gm | 23 | *g, hi, k/r, mn, o, p, q* |  |
|  |  |  | 2 | Gm | 24 | *g, hi, k/r, m/n, o, p, q* |  |
|  |  |  | 1 | Gm | 24 | *g, hi, k, mn, o, p, q, r* |  |
| 6 | settl. Chernoye (Rechitsa distr.) | 52°26'47"N; 30°22'50"E | **23** |  |  | *g, hi, k/r, m/n, o, p, q* | new data |
|  |  |  | 4 | Gm | 24 | *g, hi, k/r, m/n, o, p, q* |  |
|  |  |  | 9 | Gm | 25 | *g, hi, k, r, m/n, o, p, q,* |  |
|  |  |  | 9 | ? | 26 | *g, hi, k, m, n, o, p, q, r* |  |
|  |  |  | 1 | Sv | 25 | *g, hi, k/o, m, n, p, q, r* |  |
| 7 | settl. Krasnoye (Bragin distr.) | 51°33'50"N; 30°29'55"E | **14** |  |  |  | new data |
|  |  |  | 5 | Gm |  | *g, hi, k/r, m/n, o, p, q* |  |
|  |  |  | 3 | Gm | 25 | *g, hi, k/r, m, n, o, p, q* |  |
|  |  |  | 4 | ? | 26 | *g, hi, k, m, n, o, p, q, r* |  |
|  |  |  | 2 | Sv | 25 | *g, hi, k/o, m, n, p, q, r* |  |
| 8 | Chernobyl | 51°17'N; 30°13'E | **14** | ? | 24-26 | *–* | Baker et al. 1996 |
| 9 | Yelsk | 51°45′18″; 29°9′0″ | **5** |  |  | *g/m, h/i, k/o, n, p, q, r* | Borisov et al. 2016 |
|  |  |  | 1 | Ki | 25 | *g/m, hi, k, n, o, p, q, r* |  |
|  |  |  | 2 | Ki | 26 | *g, hi, k, m, n, o, p, q, r* |  |
|  |  |  | 2 | Ki | 27 | *g, h/i, k, m, n, o, p, q, r* |  |
| 10 | Leshnia | 52°3′20″N; 28°49′10″E | **24** |  |  | *g/m, h/i, k/o, n, p, q, r* | Borisov et al. 2016 |
|  |  |  | 1 | Ki | 25 | *g/m, h/i, k/o, n, p, q, r* |  |
|  |  |  | 2 | Ki | 25 | *g/m, hi, k, n, o, p, q, r* |  |
|  |  |  | 2 | Ki | 25 | *g, hi, k/o, m, n, p, q, r* |  |
|  |  |  | 4 | Ki | 26 | *g, hi, k, m, n, o, p, q, r* |  |
|  |  |  | 5 | Ki | 26 | *g, h/i, k/o, m, n, p, q, r* |  |
|  |  |  | 7 | Ki | 27 | *g, h/i, k, m, n, o, p, q, r* |  |
|  |  |  | 1 | 10a | 28 | *g, h, i, k, m, n, o, p, q, r* |  |
|  |  |  | 1 | Ok | 27 | *g, h/n, k, m, n, o, p, q, r* |  |
|  |  |  | 1 | Ki x Ok | 26 | *g, i/hi/hn/n, k, m, o, p, q, r* |  |
| 11 | Mosyr | 52°01′43″N; 29°19′20″E | **2** | Ki |  | *g/m, h/i, k/o, n, p, q, r* | Borisov et al. 2014 |
|  |  |  | 1 |  | 25 | *g/m, h/i, k/o, n, p, q, r* |  |
|  |  |  | 1 |  | 26 | *g, h/i, k/o, m, n, p, q, r* |  |
| 12 | Ozarichi | 52°27′49″N; 29°16′02″E | **3** | Sv |  | *g, m, h/i, k/o, n, p, q, r* | Borisov et al. 2014 |
|  |  |  | 1 |  | 26 | *g, hi, k, m, n, o, p, q, r* |  |
|  |  |  | 1 |  | 26 | *g, h/i, k/o, m, n, p, q, r* |  |
|  |  |  | 1 |  | 27 | *g, h, i, k/o, m, n, p, q, r* |  |
| 13 | Rechitsa | 52°22′28″N; 30°19′15″E | **16** |  |  |  | Borisov et al. 2016 |
|  |  |  | 3 |  | 24 | *g, hi, ko, m, n, p, q, r* |  |
|  |  |  | 4 | Sv | 25 | *g, hi, k/o, m, n, p, q, r* |  |
|  |  |  | 3 | Sv | 25 | *g, h/i, ko, m, n, p, q, r* |  |
|  |  |  | 3 | Sv | 26 | *g, h/i, k/o, m, n, p, q, r* |  |
|  |  |  | 1 | ? | 26 | *g, hi, k, m, n, o, p, q, r* |  |
|  |  |  | 1 | ? | 27 | *g, h/i, k, m, n, o, p, q, r* |  |
|  |  |  | 1 | Gm | 25 | *g, hi, k, o, m/n, o, p, q, r* |  |
|  |  |  | 2 | Sv x Gm | 24 | *g, hi, k/o, m/n, p, q, r* |  |
| 14 | Svetlogorsk (Sosnovyi Bor, west bank of the Berezina) | 52°31′46″N; 29°34′49″E | **7** | Sv |  | *g, h/i, k/o, m, n, p, q, r* | Borisov et al. 2016 |
|  |  |  | 1 | Sv | 25 | *g, hi, k/o, m, n, p, q, r* |  |
|  |  |  | 2 | Sv | 26 | *g, hi, k, m, n, o, p, q, r* |  |
|  |  |  | 1 | Sv | 26 | *g, h/i, k/o, m, n, p, q, r* |  |
|  |  |  | 3 | Sv | 27 | *g, h/i, k, m, n, o, p, q, r* |  |
| 15 | Svetlogorsk (east bank of the Berezina) | 52°39′40″N; 29°44′35″E | **3** | Sv |  | *g, h/i, k/o, m, n, p, q, r* | Borisov et al. 2016 |
|  |  |  | 1 | Sv | 26 | *g, h/i, k/o, m, n, p, q, r* |  |
|  |  |  | 1 | Sv | 26 | *g, hi, k, m, n, o, p, q, r* |  |
|  |  |  | 1 | Sv | 27 | *g, h/i, k, o, m, n, p, q, r* |  |
| 16 | Zhlobin | 52°50′32″N; 29°45′35″E | **9** | Sv |  | *g, h/i, k/o, m, n, p, q, r* | Borisov et al. 2016 |
|  |  |  | 1 | Sv | 24 | *g, hi, ko, m, n, p, q, r* |  |
|  |  |  | 2 | Sv | 26 | *g, hi, k, m, n, o, p, q, r* |  |
|  |  |  | 2 | Sv | 26 | *g, h/i, k/o, m, n, p, q, r* |  |
|  |  |  | 2 | Sv | 27 | *g, h/i, k, m, n, o, p, q, r* |  |
|  |  |  | 1 | Sv | 27 | *g, h, i, k/o, m, n, p, q, r* |  |
|  |  |  | 1 | 10a | 28 | *g, h, i, k, m, n, o, p, q, r* |  |
| 17 | Parichi | 52°48′04″N; 29°25′58″E | 13 | Sv |  |  | Borisov et al. 2016 |
|  |  |  |  | Sv | 25 | *g, hi, k/o, m, n, p, q, r* |  |
|  |  |  |  | Sv | 26 | *g, hi, k, o, m, n, p, q, r* |  |
|  |  |  |  | Sv | 26 | *g, h/i, k/o, m, n, p, q, r* |  |
|  |  |  |  | Sv | 27 | *g, h/i, k, m, n, o, p, q, r* |  |
|  |  |  | 3 | 10a | 28 | *g, h, i, k, m, n, o, p, q, r* |  |
| 18.1 | Bobruisk | 53°0′60″N; 29°12′4″E | 1 | Sv | 27 | *g, h/i, k, m, n, o, p, q, r* | Borisov et al. 2016 |
| 18.2 | Bobruisk | 53°2′N; 29°15′E | 2 | Sv | 27 | *g, h/i, k, m, n, o, p, q, r* | Mishta et al. 2000 |
| 18.3 | Bobruisk | 53°4′12”N; 29°14′28 ″E | **8** |  |  |  | new data |
|  |  |  | 2 | Sv | 25 | *g, hi, k/o, m, n, p, q, r* |  |
|  |  |  | 2 | Sv | 26 | *g, h/i, k/o, m, n, p, q, r* |  |
|  |  |  | 3 | Sv | 26 | *g, hi, k, m, n, o, p, q, r* |  |
|  |  |  | 1 | Sv | 27 | *g, h, i, k/o, m, n, p, q, r* |  |
| 19 | Bialystok | 53°6'20"N,23°9'25"E | **56** |  |  |  | Banaszek et al. 2009 |
|  |  |  | 25 | Bi | 20 | *gr, hn, ik, mp, o, q* |  |
|  |  |  | 16 | Bi | 21 | *gr, hn, ik, m/p, o, q* |  |
|  |  |  | 4 | Bi | 21 | *g/r, hn, ik, mp, o, q* |  |
|  |  |  | 6 | Bi | 22 | *g/r, hn, ik, m/p, o, q* |  |
|  |  |  | 5 | Bi | 22 | *gr, hn, ik, m, p, o, q* |  |
| 20 | Grodno | 53°38'42"N, 23°54'11"E | **1** | Bi | 22 | *gr, hn, ik, mp, o, q* | Borisov et al. 2014 |
| 21 | Lesnoe Ozero | 53°37'58"N,24°28'31"E, | **5** |  |  |  | Mishta et al. 2000 |
|  |  |  | 2 | Bi | 22 | *gr, hn, ik, m, o, p, q* |  |
|  |  |  | 2 | Bi | 23 | *g/r, hn, ik, m, o, p, q* |  |
|  |  |  | 1 | Bi | 23 | *g, hn, ik, jl, m/p, o, q, r* |  |
| 22 | Bialowieza | 52°41'41"N,23°52'38"E | **87** |  |  | *g/r, hn, ik, m/p, o, q* | Wojcik et al. 1996 |
|  |  |  | 78 | Bi | 20 | *gr, hn, ik, mp, o, q* |  |
|  |  |  | 5 | Bi | 21 | *gr, hn, ik, m/p, o, q* |  |
|  |  |  | 3 | Bi | 21 | *g/r, hn, ik, mp, o, q* |  |
|  |  |  | 1 | Bi | 22 | *gr, hn, ik, m, p, o, q* |  |
| 23 | Shatskie Ozera | 51°28'58"N,23°47'15"E | 1 | Bi |  | *gr, hn, ik, m/p, o, q* | Mishta et al. 2000 |
| 24 | Ganzevichi | 52°45'35"N; 26°24'18″E | **2** |  |  | *g/r, hn, ik, m/p, o, q* | Borisov et al. 2014 |
|  |  |  | 1 | Bi | 22 | *g/r, hn, ik, jl, m/p, o, q* |  |
|  |  |  | 1 | Bi | 23 | *g, hn, ik, jl, m/p, o, q, r* |  |
| 25 | Chervonoye | 52°22′37″N; 28°00′02″E | **15** |  |  |  | Borisov et al. 2014 |
|  |  |  | 1 | Ok | 24 | *g/r, hn, i/k, m, o, p, q* |  |
|  |  |  | 4 | Ok | 24 | *g, hn, ik, m, o, p, q, r* |  |
|  |  |  | 1 | Ok | 25 | *g, hn, i/k, m, o, p, q, r* |  |
|  |  |  | 2 | Ok | 25 | *g, h/n, ik, m, o, p, q, r* |  |
|  |  |  | 1 | Ok | 25 | *g, h/n, i/k, m/p, o, q, r* |  |
|  |  |  | 5 | Ok | 26 | *g, h/n, i/k, m, o, p, q, r* |  |
|  |  |  | 1 | Ok | 26 | *g, h, ik, m, n, o, p, q, r* |  |
|  |  |  | 1 | Ok | 26 | *g, h, i/k, m/p, n, o, q, r* |  |
| 26 | Turov | 52°4′15″N; 27°45′48″E | **12** |  |  |  | Borisov et al. 2014 |
|  |  |  | 2 | Ok | 24 | *g, hn, ik, m, o, p, q, r* |  |
|  |  |  | 1 | Ok | 25 | *g, hn, i/k, m, n, o, p, q, r* |  |
|  |  |  | 2 | Ok | 25 | *g, h/n, ik, m, n, o, p, q, r* |  |
|  |  |  | 1 | Ok | 25 | *g, h/n, i/k, m/p, o, q, r* |  |
|  |  |  | 1 | Ok | 26 | *g, h/n, i/k, m, n, o, p, q, r* |  |
|  |  |  | 2 | Ok | 27 | *g, h/n, i, k, m, n, o, p, q, r* |  |
|  |  |  | 2 | 10a | 28 | *g, h, i, k, m, n, o, p, q, r* |  |
|  |  |  | 1 | Sv x Ok | 26 | *g, h, i/ik/ko/o, m, n, p, q, r* |  |
|  |  |  | **9** |  |  |  | Borisov et al. 2016 |
|  |  |  | 2 | Ok | 24 | *g, hn, ik, m, n, o, p, q, r* |  |
|  |  |  | 2 | Ok | 25 | *g, h/n, ik, m, n, o, p, q, r* |  |
|  |  |  | 2 | Ok | 26 | *g, h/n, i/k, m, n, o, p, q, r* |  |
|  |  |  | 1 | Ok | 26 | *g, h, ik, m, n, o, p, q, r* |  |
|  |  |  | 2 | Ok | 27 | *g, h, i/k, m, n, o, p, q, r* |  |
| 27 | Khvoyensk | 52°2′11″N; 27°56′40″E | **8** |  |  |  | Borisov et al. 2014 |
|  |  |  | 1 | Ok | 25 | *g/r, h/n, i/k, m, o, p, q* |  |
|  |  |  | 3 | Ok | 25 | *g, hn, i/k, m, o, p, q, r* |  |
|  |  |  | 1 | Ok | 25 | *g, h/n, ik, m, o, p, q, r* |  |
|  |  |  | 1 | Ok | 27 | *g, h/n, i, k, m, o, p, q, r* |  |
|  |  |  | 1 | 10a | 28 | *g, h, i, k, m, n, o, p, q, r* |  |
|  |  |  | 1 | Sv x Ok | 25 | *g/m, h/n, i/k, o, p, q, r* |  |
|  |  |  | **13** |  |  |  | Borisov et al. 2016 |
|  |  |  | 1 | Ok | 25 | *g, hn, i/k, m, o, p, q, r* |  |
|  |  |  | 4 | Ok | 25 | *g, h/n, ik, m, o, p, q, r* |  |
|  |  |  | 3 | Ok | 26 | *g, h/n, i/k, m, o, p, q, r* |  |
|  |  |  | 1 | Ok | 26 | *g, h, ik, m, n, o, p, q, r* |  |
|  |  |  | 3 | Ok | 27 | *g, h, i/k, m, n, o, p, q, r* |  |
|  |  |  | 1 | 10a | 28 | *g, h, i, k, m, n, o, p, q, r* |  |
| 28 | Konkovichi | 52°9′22″N, 28°43′30″E | **6** |  |  |  | Borisov et al. 2014 |
|  |  |  | 2 | Ok |  | *g, h/n, i/k, m, o, p, q, r* |  |
|  |  |  | 4 | 10a |  | *g, h, i, k, m, n, o, p, q, r* |  |
| 29.1 | Oktiabr’skiy, west bank of the Ptich | 52°34′26″N; 28°44′37″E | **9** |  |  |  | Borisov et al. 2014 |
|  |  |  | 2 | Ok | 25 | *g, hn, i/k, m, o, p, q, r* |  |
|  |  |  | 1 | Ok | 25 | *g, h/n, ik, m, o, p, q, r* |  |
|  |  |  | 2 | Ok | 26 | *g, h/n, i/k, m, o, p, q, r* |  |
|  |  |  | 2 | Ok | 27 | *g, h/n, i, k, m, o, p, q, r* |  |
|  |  |  | 2 | 10a | 28 | *g, h, i, k, m, n, o, p, q, r* |  |
|  |  |  | 1 | Sv | 26 | *g, hi, i, k, m, o, p, q, r* |  |
|  |  |  | **4** |  |  |  | Borisov et al. 2016 |
|  |  |  | 1 | Ok | 24 | *g, hn, ik, m, o, p, q, r* |  |
|  |  |  | 1 | Ok | 27 | *g, h/n, i, k, m, o, p, q, r* |  |
|  |  |  | 1 | Sv | 26 | *g, hi, i, k, m, o, p, q, r* |  |
|  |  |  | 1 | Sv x Ok | 26 | *g, h, i/ik/ko/o, m, n, p, q, r* |  |
| 29.2 | Oktiabr’skiy, east bank of the Ptich | 52°35′51″N; 28°45′08″E | **9** |  |  |  | Borisov et al. 2014 |
|  |  |  | 2 | Ok | 25 | *g, hn, i/k, m, o, p, q, r* |  |
|  |  |  | 1 | Ok | 25 | *g, h/n, ik, m, o, p, q, r* |  |
|  |  |  | 2 | Ok | 26 | *g, h/n, i/k, m, o, p, q, r* |  |
|  |  |  | 2 | Ok | 27 | *g, h/n, i, k, m, o, p, q, r* |  |
|  |  |  | 2 | 10a | 28 | *g, h, i, k, m, n, o, p, q, r* |  |
|  |  |  | **19** |  |  |  | Borisov et al. 2016 |
|  |  |  | 2 | Ok | 25 | *g, h/n, ik, m, o, p, q, r* |  |
|  |  |  | 4 | Ok | 26 | *g, h/n, i/k, m, o, p, q, r* |  |
|  |  |  | 4 | Ok | 27 | *g, h/n, i, k, m, o, p, q, r* |  |
|  |  |  | 2 | Ok | 27 | *g, h, i/k, m, n, o, p, q, r* |  |
|  |  |  | 6 | 10a | 28 | *g, h, i, k, m, n, o, p, q, r* |  |
|  |  |  | 1 | Sv x Ok | 26 | *g, h/n, i, k/o, m, q, r* |  |
| 30 | Tatarka | 53°15′22″N; 28°48′24″E | **13** |  |  |  | Borisov et al. 2016 |
|  |  |  | 6 | Ok | 24 | *g, hn, ik, m, o, p, q, r* |  |
|  |  |  | 4 | Ok | 25 | *g, hn, i/k, m, o, p, q, r* |  |
|  |  |  | 1 | Ok | 25 | *g, h/n, i/k, m/p, o, q, r* |  |
|  |  |  | 3 | Ok | 26 | *g, h/n, i/k, m, o, p, q, r* |  |
| 31 | settl. Elizovo (Bobruisk distr.) | 53°24′20″N; 29°0′30″E | **4** |  |  | *g, h/n, i/k, m, o, p, q, r* | new data |
|  |  |  |  | Ok | 25 | *g, hn, i/k, m, o, p, q, r* |  |
|  |  |  |  | Ok | 25 | *g, h/n, ik, m, o, p, q, r* |  |
|  |  |  |  | Ok | 26 | *g, h/n, i/k, m, o, p, q, r* |  |
| 32 | settl. Lyubonichi (Bobruisk Distr.) | 53°15′19″N; 29°10′21″E | **14** | Ok |  | *g, h/n, i/k, m, o, p, q, r* | new data |
|  |  |  | 2 | Ok | 25 | *g, hn, i/k, m, o, p, q, r* |  |
|  |  |  | 2 | Ok | 25 | *g, h/n, ik, m, o, p, q, r* |  |
|  |  |  | 5 | Ok | 26 | *g, h/n, i/k, m, o, p, q, r* |  |
|  |  |  | 1 | Ok | 27 | *g, h/n, i, k, m, o, p, q, r* |  |
|  |  |  | 2 | Ok | 27 | *g, h, i/k, m, n, o, p, q, r* |  |
|  |  |  | 2 | 10a |  | *g, h, i, k, m, n, o, p, q, r* |  |
| 33 | Kardymovo (Smolensk region, RF) | 54°55'N; 32°15'E | 2 | Wd | 18 | *gm, hk, ip, no, qr* | Orlov and Borisov 2009 |
| 34 | Dubrovno | 54°35′N; 31°00′E | **3** |  |  |  | Orlov, Borisov 2009 |
|  |  |  | 2 | Wd | 19 | *g/m, hk, ip, no, qr* |  |
|  |  |  | 1 | Wd | 21 | *g/m, hk, i/p, no, q/r* |  |
| 35 | Smolyany | 54°35′N; 30°00′E | 2 | Bs | 22 | *g/m, hk, i, no, p, q/r* | Orlov, Borisov 2009 |
| 36 | Malyi Vyazok | 54°10'0''N; 29°11'25''E | **3** |  |  | *g/m, h/k, i, n/o, p, q/r* | Orlov, Borisov 2009 |
|  |  |  |  | Bs | 24 | *g/m, hk, i, n/o, p, q, r* |  |
|  |  |  |  | Bs | 25 | *g/m, h/k, i, n/o, p, q, r* |  |
|  |  |  |  | Bs | 25 | *g/m, hk, i, n, o, p, q, r* |  |
|  |  |  | **24** |  |  |  | Borisov et al. 2010 |
|  |  |  | 5 | Bs | 22 | *gm, hk, i, n/o, p, q/r* |  |
|  |  |  | 4 | Bs | 23 | *gm, hk, i, n/o, p, q, r* |  |
|  |  |  | 4 | Bs | 24 | *gm, hk, i, n, o, p, q, r* |  |
|  |  |  | 3 | Bs | 24 | *g/m, hk, i, n/o, p, q, r* |  |
|  |  |  | 1 | Bs | 24 | *gm, h/k, i, n/o, p, q, r* |  |
|  |  |  | 1 | Bs | 26 | *g/m, h/k, i, n, o, p, q, r* |  |
|  |  |  | 1 | Bs | 24 | *g, hk, i, m, no, p, q, r* |  |
|  |  |  | 5 | Bs | 25 | *g/m, hk, i, n, o, p, q, r* |  |
| 37 | Vydriza | 54°10'32"N; 29° 2'59"E | **4** |  |  |  | Borisov et al. 2010 |
|  |  |  | 1 | Bs | 22 | *gm, hk, i, n/o, p, q/r* |  |
|  |  |  | 1 | Bs | 23 | *gm, hk, i, n/o, p, q, r* |  |
|  |  |  | 2 | Bs | 24 | *gm, hk, i, n, o, p, q, r* |  |
| 38 | Velyatichi | 54°9'45'E'; 28°54'54''E | **2** |  |  | *gm, hk, i, n/o, p, q/r* | Borisov et al. 2010 |
|  |  |  | 1 | Bs | 22 | *gm, hk, i, n/o, p, q/r* |  |
|  |  |  | 1 | Bs | 23 | *gm, hk, i, n/o, p, q, r* |  |
| 39 | Novaya Metcha | 54°11'10''N, 28°43'41'' E | **2** | Bs | 23 | *gm, hk, i, n/o, p, q, r* | Orlov, Borisov 2009 |
|  |  |  | **4** | Bs | 23 | *gm, hk, i, n/o, p, q, r* | Borisov et al. 2010 |
| 40 | Leskovichi | 53°55'48''N; 29°7'41''E | **2** |  |  |  | Borisov et al. 2010 |
|  |  |  | 1 | ? | 24 | *gm, hk, i, n, o, p, q, r* |  |
|  |  |  | 1 | ? | 25 | *g/m, hk, i, n, o, p, q, r* |  |
| 41 | Mikhevichi | 54°1'20''N; 29°9'57''E | **5** |  |  | *g/m, h/k, i, n, o, p, q, r* | Borisov et al. 2010 |
|  |  |  | 2 | ? | 25 | *gm, h/k, i, n, o, p, q, r* |  |
|  |  |  | 2 | ? | 25 | *g/m, hk, i, n, o, p, q, r* |  |
|  |  |  | 1 | ? | 26 | *g, hk, i, m, n, o, p, q, r* |  |
| 42 | Berezino, east bank of the Berezina | 53°49'13''N; 29°0'23''E | **10** |  |  | *g, m, h/k, i, n, o, p, q, r* | Borisov et al. 2010 |
|  |  |  |  | ? | 26 | *g, hk, i, m, n, o, p, q, r* |  |
|  |  |  |  | ? | 27 | *g, h/k, i, m, n, o, p, q, r* |  |
|  |  |  |  | 10a | 28 | *g, h, i, k, m, n, o, p, q, r* |  |
| 43 | Yedlino, west bank of the Berezina | 53°44'18''N; 28°56'16''E | **2** | ? |  | *g, h/k, i, m, n, o, p, q, r* | Orlov, Borisov 2009 |
|  |  |  | **17** |  |  |  | Borisov et al. 2010 |
|  |  |  | 1 | ? | 25 | *g/m, hk, i, n, o, p, q, r* |  |
|  |  |  | 6 | ? | 26 | *g, hk, i, m, n, o, p, q, r* |  |
|  |  |  | 6 | ? | 27 | *g, h/k, i, m, n, o, p, q, r* |  |
|  |  |  | 4 | 10a | 28 | *g, h, i, k, m, n, o, p, q, r* |  |
